# Large Parotid Gland Lipoblastoma in a Teenager

**DOI:** 10.3389/fped.2018.00050

**Published:** 2018-03-09

**Authors:** Danny Jandali, Ashley Heilingoetter, Ritu Ghai, Jill Jeffe, Samer Al-Khudari

**Affiliations:** ^1^Department of Otorhinolaryngology – Head and Neck Surgery, Rush University Medical Center, Chicago, IL, United States; ^2^Rush Medical College, Rush University Medical Center, Chicago, IL, United States; ^3^Department of Pathology, Rush University Medical Center, Chicago, IL, United States

**Keywords:** lipoblastoma, parotid gland, head and neck mass, parotid gland tumor, facial nerve

## Abstract

**Background:**

Lipoblastomas are rare benign neoplasms that arise from fetal white fat cells. They are typically found in children under the age of 3 and have been reported in the mediastinum, extremities, and infrequently in the head and neck. We present a rare case of a lipoblastoma arising from the parotid gland and the first known report of a parotid lipoblastoma in a teenager.

**Case presentation:**

A 15-year-old male presented with a painless, slowly enlarging parotid mass and left facial swelling. A fine needle aspiration was non-diagnostic and initial MRI showed a 3.8 cm × 5.0 cm × 4.0 cm fatty lesion involving the superficial and deep lobes of the left parotid gland and masticator space with widening of the stylo-mandibular tunnel and thinning of the adjacent mandibular condyle. The patient was taken to the operating room, and the mass was excised under general anesthesia *via* a transcervical parotid approach with facial nerve monitoring. The most superficial aspect of the parotid bed was spared and with upper and lower divisions of the facial nerve preserved. The tumor, which primarily involved the deep lobe of the parotid, was entirely excised. Final pathology revealed a 5.2 cm lipoblastoma. The patient did well post-operatively with full function of the facial nerve and 20 months of follow up without evidence of recurrence.

**Conclusion:**

This is the first reported case of a lipoblastoma of the parotid gland in a teenager. Although a rare tumor, it should be considered in the differential diagnosis of a parotid mass in this population.

## Background

Lipoblastoma is a rare, rapidly growing, benign neoplasm of embryonal white fat (1–3). These tumors are most often seen in male children under the age of 3, with a median age at diagnosis of 1 year (3, 4). Lipoblastoma can be found anywhere in the body, but it is typically located in the extremities, trunk, mediastinum, and less frequently in the head and neck (1, 3). Within the head and neck, the cervical region is the most common site of presentation, with the salivary glands and parapharyngeal space being other notable locations (2). While these lesions are benign, they have the propensity to recur, requiring multiple excisions (5). We present a rare case of a lipoblastoma arising from the parotid gland and the first known report of a parotid lipoblastoma in a teenager.

## Case Presentation

A 15-year-old male with a history of asthma presented to the Rush University Otorhinolaryngology clinic with a 1-year history of a painless, slowly enlarging left parotid gland mass and left-sided facial swelling. He denied dysphagia, difficulty breathing, or facial paresis. Physical examination was significant for a 4-cm spongy, non-fixed mass of the left parotid gland at the lobule of the ear.

**Figure 1:**
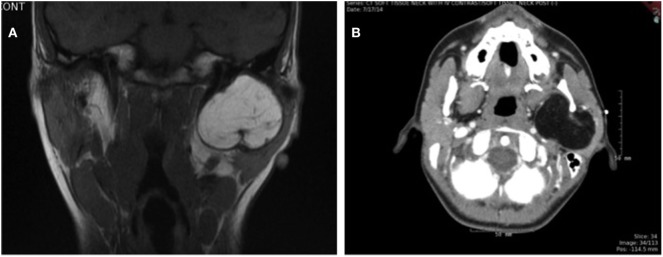
**(A)** T1-weighted coronal MRI image and **(B)** axial CT image showing large mass in left parotid gland.

Initial ultrasound demonstrated calcifications, and a fine needle aspiration was non-diagnostic. MRI soft tissue neck with and without contrast showed a 3.8 cm × 5.0 cm × 4.0 cm fatty lesion involving the superficial and deep lobes of the left parotid gland and masticator space with widening of the stylo-mandibular tunnel, with the tumor abutting the medial surface of ramus of the mandible (Figure 1A). The patient underwent a preoperative CT soft tissue neck with contrast, showing a dumbbell-shaped hypodense lesion in the deep lobe of the parotid gland, with anterior displacement of the pterygoids and medial displacement of the parapharyngeal space, as well as mild bony remodeling of the mandibular ramus and thinning of the mandibular condyle (Figure 1B).

**Figure 2:**
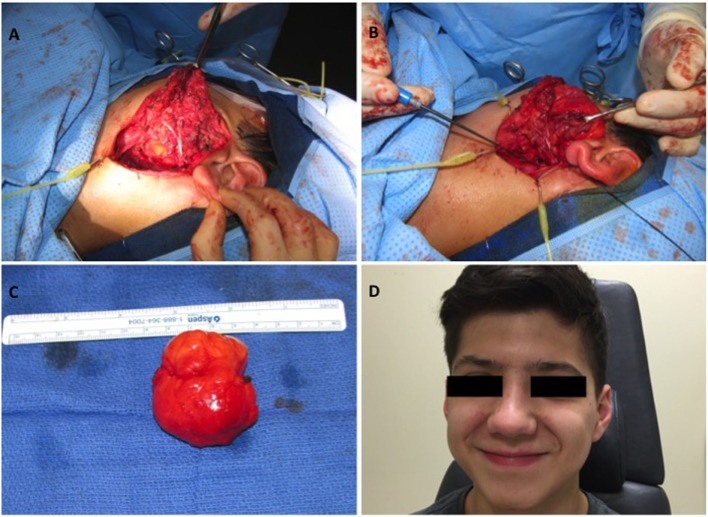
Intraoperative and postoperative photos: **(A)** lipoblastoma in relation to the deep lobe of the parotid gland and the upper and lower divisions of the facial nerve, **(B)** lipoblastoma prior to excision from parotid gland, **(C)** excised parotid gland lipoblastoma (5.2 cm × 4.3 cm × 3.5 cm), **(D)** No evidence of tumor recurrence at 20 months of follow up.

**Figure 3:**
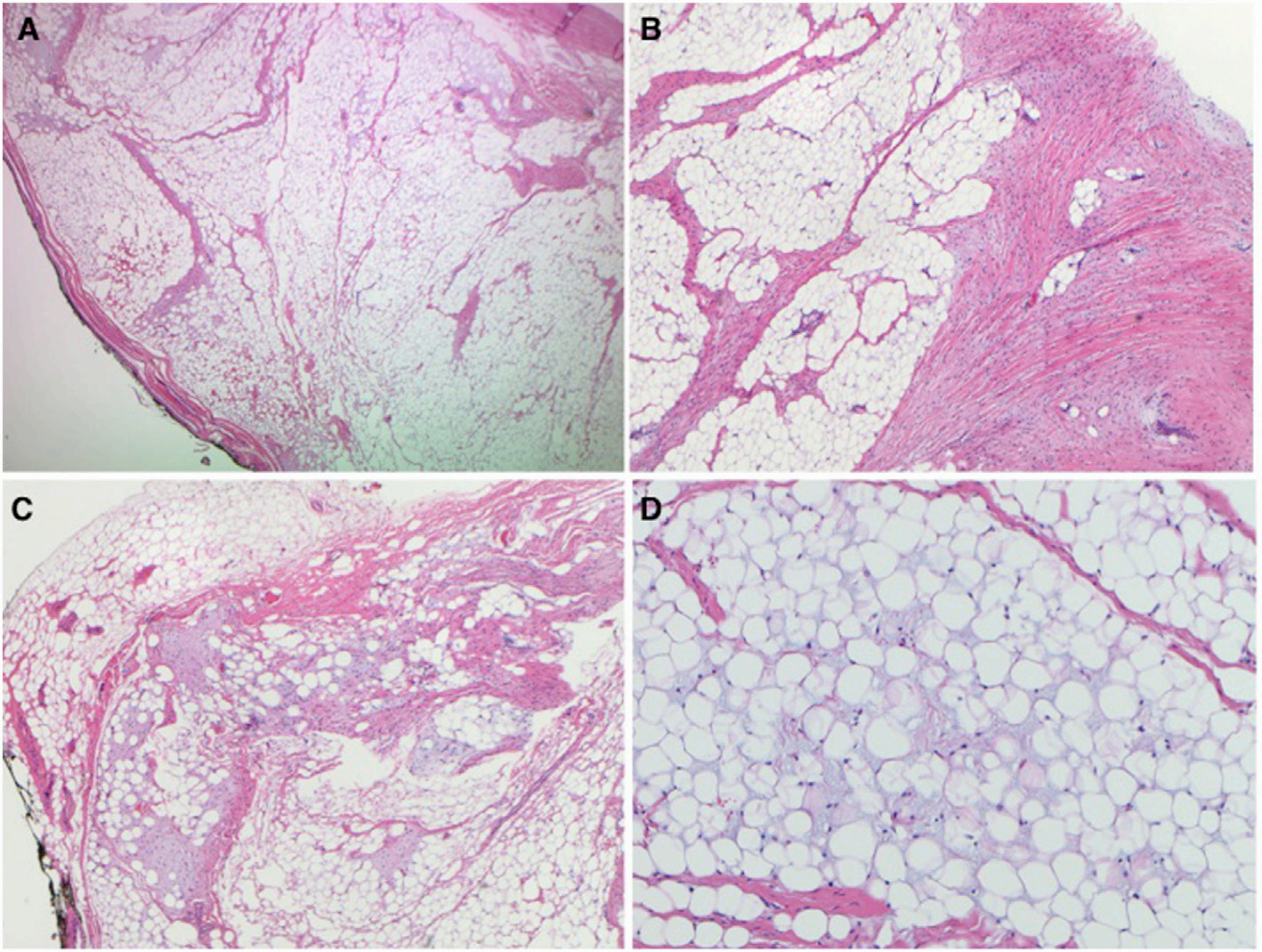
Pathology images: **(A)** well-defined nodule with fibrous septae (2×), **(B)** intervening white fibrous trabeculae (4×), **(C)** myxoid stroma at the periphery of the nodule (4×), **(D)** mature adipose tissue with myxoid material in the background (10×).

The patient was taken to the operating room, and the mass was excised *via* a transcervical parotid approach under general anesthesia with facial nerve monitoring. The superficial aspect of the parotid bed was spared, and upper and lower divisions of the facial nerve were identified and preserved (Figure 2A). The tumor, which primarily involved the deep lobe of the parotid gland, was entirely excised (Figures 2B,C). Reactive level II nodes were identified preoperatively on imaging. Intraoperatively a 3cm × 1.6 cm level II node was identified and subsequently excised. Final pathology revealed a 5.2 cm × 4.3 cm × 3.5 cm lipoblastoma and one left cervical lymph node with reactive hyperplasia. The lipoblastoma was described as a well-defined mass composed predominantly of lobules of mature adipose tissue with fibrous septae (Figures 3A,B). Areas of myxoid stroma were noted at the periphery of the mass with nodules and strands of fibrous tissue within the center of the mass (Figures 3C,D). Rare lipoblasts were identified, with no evidence of cytologic atypia and no mitosis to suggest malignant transformation.

The patient recovered well post-operatively, aside from a brief spell of first bite syndrome that ultimately resolved. He has full function of the facial nerve, with intact upper and lower divisions, and 20 months of follow-up without evidence of recurrence (Figure 2D).

## Discussion

Lipoblastoma is a rare, benign neoplasm of embryonal white fat (1, 3). It is most often seen in children under the age of 3 and is extremely rare in children older than 8 years, making the case presented here particularly noteworthy (3–5). Lipoblastoma has a predilection for males, with sources citing as high as a 4:1 ratio of affected males to females (1, 5). The typical presentation of lipoblastoma of the head and neck is a chronic, painless, progressively enlarging mass. Other symptoms, depending on location with the head and neck, include respiratory distress and muscle weakness from nerve compression (1, 2). Although our patient’s tumor was within the parotid gland parenchyma, facial nerve function was not affected. The parotid gland is a relatively uncommon location for lipoblastoma to develop, with a total of seven cases described in the literature, including this one (Table 1).

**Table 1:** Reported cases of parotid gland lipoblastoma.

Author(s)	Patient’s age	Patient’s gender	Tumor size (cm)	Treatment	Follow-up
Calhoun et al. (6)	7 months	Male	3.0 × 3.0	Complete surgical excision	N/A
Krempl et al. (7)	13 months	Male	3.0 × 2.7 × 0.5	Complete surgical excision	No recurrence at 6-month follow up
Leon et al. (8)	6 years	Male	7.0 × 6.0 × 3.5	Complete surgical excision	N/A
Matsushima et al. (9)	2 years	Female	N/A*	Complete surgical excision	N/A*
Anantharajan and Ravindranathan (4)	3 years	Female	11.0 × 8.0	Complete surgical excision	No recurrence at 3-year follow-up
Xie and Kubba (10)	6 months	Male	N/A	Complete surgical excision	N/A
Our case	15 years	Male	5.2 × 4.3 × 3.5	Complete surgical excision	No recurrence at 20-month follow-up

*This article is written in Japanese.

In comparison to other reported cases of parotid gland lipoblastoma, our patient was substantially older at age 15, with the next oldest patient being 6 years of age (4, 6–10). Our patient was male, which is similar to most reported cases (71%) and consistent with the literature on these tumors (1, 5). Many case reports did not disclose follow-up time after surgical excision. The present case was followed for 20 months, allowing substantial time to assess for recurrence. Of the five cases for which size was recorded, the average tumor size was 5.8 cm, slightly larger than our patient’s tumor at 5.2 cm (4, 6–8). All reported cases of parotid gland lipoblastoma underwent complete surgical excision. Among case reports that disclosed it, the type of surgical approach varied based on the tumor location and extension. One patient underwent a total parotidectomy, while the rest had only a portion of the parotid gland removed, depending on the location of the tumor within the parotid gland (4, 7, 8). All cases were reported to have intact facial nerve function after surgery, including our patient.

Differential diagnosis includes lipoblastoma, lipoma, teratoma, and myxoid liposarcoma, with the most important alternative diagnosis to consider being myxoid liposarcoma (1). Myxoid liposarcoma and lipoblastoma cannot be differentiated on imaging; however, they can be differentiated histologically or *via* cytogenetic analysis. Liposarcomas are extremely rare in patients under 10 years of age (1). Given that this patient was 15 years old at the time of diagnosis, the possibility of liposarcoma was considered. However, fine needle aspiration did not yield malignant cells.

Grossly, lipoblastomas range in size from 3 to 12 cm (2). Histologically, these neoplasms comprise lipoblasts—i.e., immature fat cells—in varying stages of maturity, as well as primitive mesenchymal cells, myxoid matrix, and a plexiform capillary network, all of which is organized into lobules *via* fibrous stroma (1, 3). The presence of nonfat components differentiates lipoblastoma from lipoma. Lipoblastoma is a benign tumor and shows no pleomorphism or anaplasia (5).

In the literature, lipoblastoma is often noted to have a lobular appearance with internal septations on imaging (1). Its appearance on imaging depends on the proportion of fat relative to myxoid stroma. The fat components appear hyperechogenic on ultrasound, confer low attenuation on CT images, and demonstrate high signal intensity on T1- and T2-weighted MRI images (1). Alternatively, the myxoid stroma components of the neoplasm are hypoechoic on ultrasound, have low attenuation on CT images but are less hypodense than fat, and demonstrate low signal intensities on T1-weighted MRI images and high signal intensities on T2-weighted MRI images (1). The patient presented here underwent ultrasound, CT, and MRI imaging prior to surgery. Subtle characteristics seen on MRI may have been missed due to interference from dental braces. CT findings were significant for mass effect of this lesion, but no major differences in attenuation were noted, making this difficult to differentiate from a lipoma preoperatively. It is conceivable that, for many patients, suspicion of lipoblastoma may not be raised until the mass is surgically removed.

These neoplasms are categorized into two types histologically: encapsulated and diffuse. Complete surgical excision of these tumors can lead to cure; however, they often recur. Several authors have suggested the diffuse type of lipoblastoma tends to be more commonly associated with recurrence (3). Our patient’s tumor was encapsulated, and accordingly, he fortunately has yet to demonstrate recurrence of this tumor after 20 months of follow up.

## Concluding Remarks

In summary, head and neck lipoblastoma is a rare benign tumor seen in young children, most often under the age of 3. In the head and neck, it typically presents as a painless, progressively enlarging mass, with the potential to cause airway obstruction or nerve compression. Subtle differences in imaging characteristics may allow this mass to be differentiated from a lipoma; however, this was not the case for our patient. This mass was suspected to be a lipoma until the final pathology report confirmed it as lipoblastoma, an uncommon finding in this age group. This is the first known reported case of a lipoblastoma of the parotid gland in a teenager. Although a rare tumor, it should be considered in the differential diagnosis of a parotid mass in this population.

## Informed Consent

Written informed consent was obtained from the patient’s parent for publication of this case report.

## Ethics Statement

Ethical consideration was given to the present case. This is a case report and does not require committee approval. Parental consent was obtained for this case, and patient confidentiality was maintained in the reporting of this case.

## Author Contributions

DJ, AH, JJ, and SA-K contributed to writing and editing the abstract and case report. RG reviewed relevant histology and provided photos and captions for this work. All authors approved of the final submitted version of this document.

## Conflict of Interest Statement

The authors declare that the research was conducted in the absence of any commercial or financial relationships that could be construed as a potential conflict of interest.
